# Femtosecond Laser-Assisted In Situ Keratomileusis Treatment of Residual Refractive Error following Femtosecond Laser-Enabled Keratoplasty

**DOI:** 10.1155/2019/8520183

**Published:** 2019-08-29

**Authors:** Elizabeth Shen, Lester Tsai, Hannah Muniz Castro, Matthew Wade, Marjan Farid

**Affiliations:** Gavin Herbert Eye Institute, University of California Irvine School of Medicine, Irvine, CA 92697, USA

## Abstract

**Purpose:**

To evaluate the safety and effectiveness of femtosecond laser-assisted in situ keratomileusis (LASIK) in the treatment of residual myopia and astigmatism following femtosecond laser-enabled keratoplasty (FLEK).

**Design:**

Retrospective case review.

**Methods:**

Chart review of all patients with prior FLEK who subsequently underwent femto-LASIK surgery after full suture removal was performed at the Gavin Herbert Eye Institute at the University of California, Irvine. A total of 14 eyes in 13 patients met this criterion, and their comprehensive examinations performed at standard intervals were reviewed. Main outcome measures include uncorrected distance visual acuity (UDVA) and corrected distance visual acuity (CDVA) expressed as the logarithm of the minimum angle of resolution (logMAR), manifest refractive astigmatism, and spherical equivalent.

**Results:**

From the preoperative visit to the 3 month visit, all 14 eyes significantly improved in UDVA (logMAR, 0.93 ± 0.23 to 0.44 ± 0.32, *P* = 0.002) with no loss of CDVA (logMAR, 0.26 ± 0.19 to 0.18 ± 0.23, *P* = 0.50). All 14 eyes showed significant improvement in manifest refractive astigmatism (4.71 ± 1.77 to 2.18 ± 1.45 diopters (D), *P* = 0.003) and spherical equivalent (−2.57 ± 2.45 to −0.48 ± 0.83 D, *P* = 0.0007). There were no flap or graft complications as a result of femto-LASIK.

**Conclusions:**

Our findings suggest that femto-LASIK on eyes with prior FLEK is safe and effective in improving visual acuity and reducing residual astigmatism.

## 1. Introduction

In penetrating keratoplasty (PK), a variety of perioperative factors influences the refractive outcome, which makes it challenging to achieve a desirable and predictable result [1]. These include, but are not limited to, the preoperative state of the diseased host tissue, quality of the donor tissue, intraoperative handling of the graft, and postoperative tissue healing [2]. Furthermore, despite advances in microsurgical techniques, the success of conventional PK with blade trephination is often compromised by poor visual acuity and astigmatism. Most mild to moderate cases of residual refractive error can simply be corrected with spectacles or contact lenses. However, the anisometropia and astigmatism from larger refractive errors may not be as tolerable [3]. In these cases as well as ones in which contact lens wear is not tolerated, corrective refractive surgery is a viable option to achieve emmetropia.

After the graft has healed and stabilized, a variety of corneal and lenticular surgeries may be performed to neutralize refractive error [1]. Intraocular surgeries include phakic or pseudophakic intraocular lens and refractive lens exchange. Corneal surgeries include astigmatic keratotomy, intrastromal corneal ring segments, photorefractive keratectomy, and laser-assisted in situ keratomileusis (LASIK) [1]. Among these procedures, femtosecond LASIK, or femto-LASIK, is the most preferred and most common post-PK corrective surgery [4, 5]. Its popularity stems from its ability to correct a wide range of refractive errors with reportedly better and more predictable visual recovery outcomes than other procedures [2, 6–8]. Complications are relatively low in incidence but include corneal perforation and scarring, flap-related issues, epithelial ingrowth, and graft-host junction dehiscence [2, 8, 9]. Taken together, femto-LASIK has become a reasonable option for correcting residual refractive errors from PK.

The advent of femtosecond (FS) laser improved PK surgeries by creating precise and consistent incisions identical in host and donor corneas [10]. Compared to traditional PK with manual blade trephination, femtosecond laser-enabled keratoplasty (FLEK) results in similar and often better visual recovery and astigmatism [11–17]. Nevertheless, severe cases of post-FLEK anisometropia and astigmatism can still occur. Given the successful refractive outcomes from femto-LASIK on eyes with prior conventional PK, it is reasonable to expect similar, if not better, outcomes for femto-LASIK on eyes with prior FLEK. Currently, to the best of our knowledge, there are no studies reporting outcomes of femto-LASIK after FLEK. In this study, we report the results of patients who underwent femto-LASIK on eyes with prior FLEK.

## 2. Materials and Methods

### 2.1. Patient Selection

Patients were included in this study if they had a prior FLEK with clear and stable grafts after full suture removal and a subsequent LASIK surgery from September 2008 to July 2016 at the Gavin Herbert Eye Institute, University of California, Irvine. These patients were also included regardless of other corneal or lenticular surgeries that may have occurred in between FLEK and femto-LASIK surgeries, including cataract extraction and intraocular lens placement or intraocular lens exchange and repositioning.

### 2.2. Evaluation and Data Collection

Preoperatively, each patient was screened for refractive stability and for retinal pathologies, glaucoma, and other ocular diseases that could affect visual potential. These complete ophthalmic examinations included measurements of uncorrected distance visual acuity (UDVA) and corrected distance visual acuity (CDVA) with a Snellen chart, intraocular pressure, manifest refraction, and corneal topography with the Pentacam (Oculus, Wetzlar, Germany), ATLAS (Carl Zeiss Meditec, Inc., Dublin, CA), Orbscan (Bausch & Lomb, Rochester, NY), and the IOLMaster (Carl Zeiss Meditec, Inc., Dublin, CA). Refractive stability was ensured postsuture removal by at least three serial refraction and topographies prior to a final decision on laser refractive correction. Graft health was primarily determined by endothelial cell analysis using specular microscopy with a CellChek XL (Konan, Irvine, CA). Complete ophthalmic examinations were also performed routinely at postoperative months 1, 3, 12, and beyond, up to 3 years. Given the visits that occurred 1 to 3 years postoperatively varied in time and number, the patient's last visit during that time period was chosen as the “1 yr+” visit. The “Final” visit represents the last follow-up visit that occurred within those 3 years. Data collection at routine follow-up visits included UDVA, CDVA, and manifest refraction. Corneal topography was also assessed when available. Institutional Review Board approval was obtained at the University of California, Irvine, for this retrospective study.

### 2.3. Operative Technique

All FLEK and femto-LASIK surgeries were performed at Gavin Herbert Eye Institute at University of California, Irvine. All surgeries followed standard protocol. Two surgeons (RFS and MF) performed the surgeries using the same techniques. Patients reviewed and signed informed consent forms prior to each surgery.

FLEK surgeries were performed with the IntraLase FS laser (Johnson & Johnson Surgical Vision, Santa Ana, CA). Identical zig-zag patterns were used to create full-thickness cuts in host and donor corneas. Three separate cuts were created to intersect contiguously in order to form a full-thickness cut from the corneal surface to the anterior chamber. The first cut extends from the posterior corneal surface in the anterior chamber into the stroma at an angle of 30° towards the periphery. The second cut consists of a lamellar ring with a width of 0.5 mm at a depth of 320 *μ*m from the anterior corneal surface. The final cut extends from the anterior corneal surface into the stroma at an angle of 30° towards the periphery. The anterior diameter of the cut in each host cornea was set at 8, 8.5, or 9 mm, and the graft size was accordingly set at an identical diameter. Standard postoperative drops included moxifloxacin and prednisolone acetate 1%, tapered per surgeon preference. Postoperative healing of wound was ensured prior to full suture removal, which occurred postoperatively between 3 and 26 months.

In all cases, the surgeon who performed the patient's FLEK also subsequently treated the patient with femto-LASIK surgery. A 150-kHz FS laser (IntraLase FS, IntraLase Corp., Irvine, CA) was used to create all flaps. Flaps were created within the diameter of the PK graft at an intended thickness of 120 *μ*m, along with a superior hinge. No flap crossed the graft-host junction. After separating and lifting the flap, the stroma was ablated with a VISX Star S4 excimer laser (VISX, Santa Ana, CA). The ablation was set for a conventional treatment based on manifest refraction. If femto-LASIK retreatment was needed, the flap was relifted and the stromal bed was reablated. Postoperatively, patients were prescribed moxifloxacin and prednisolone acetate 1%, tapered per surgeon preference.

### 2.4. Analysis

For statistical analysis, Snellen values from UDVA and CDVA were converted to logarithm of the minimum angle of resolution (logMAR) scale. Outcome measurements of UDVA, CDVA, manifest astigmatism, and spherical equivalent were compared preoperatively and postoperatively using paired and unpaired *t* test analyses in Microsoft Excel (Microsoft Corp, Redmond, WA). A *P* value less than 0.05 was considered statistically significant.

## 3. Results

### 3.1. Patient Characteristics

Patient characteristics are presented in Table 1. Fourteen eyes (4 right, 10 left) from thirteen patients (7 male, 6 female) were included in this study. The mean age of the patient at the time of femto-LASIK was 50.8 ± 18.5 years (range, 17.6–77.4 years), and the mean time from FLEK to femto-LASIK was 25.3 ± 17.6 months (range, 7.4–69.5 months). The mean time from full suture removal of FLEK to femto-LASIK was 14.7 months (range, 2.4–57.6 months). Primary diagnosis prior to FLEK included graft failure, corneal scar, keratoconus, postsurgical ectasia, and Fuchs' dystrophy. Some patients underwent additional procedures after FLEK but prior to femto-LASIK surgery by at least 3 months. These included cataract exchange and intraocular lens placement as well as intraocular lens exchange and repositioning. Of 13 total subjects, 10 were examined at 1-month follow-up, 10 at 3-month follow-up, 8 at 12-month follow-up, and 5 at follow-up exams thereafter.

### 3.2. Visual Acuity

**Figure 1:**
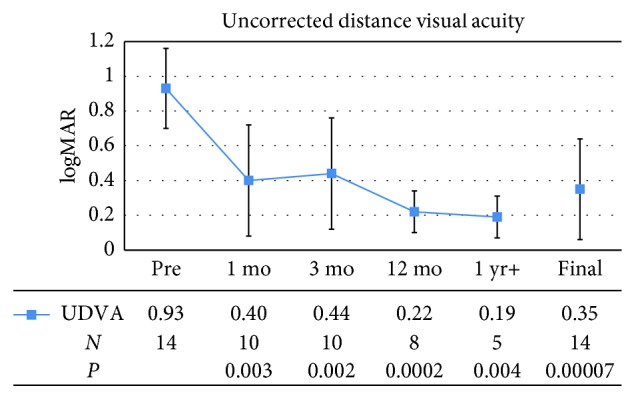
Mean preoperative (Pre) and postoperative uncorrected distance visual acuity (UDVA), expressed as the logarithm of the minimum angle of resolution (logMAR). Error bars represent one standard deviation. mo = month; yr = year; *N* = number of patients.

**Figure 2:**
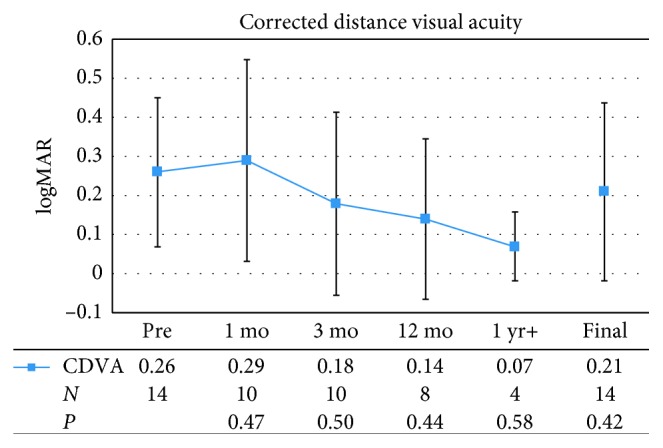
Mean preoperative (Pre) and postoperative corrected distance visual acuity (CDVA), expressed as the logarithm of the minimum angle of resolution (logMAR). Error bars represent one standard deviation. mo = month; yr = year; *N* = number of patients.

Figures 1 and 2 demonstrate the mean UDVA and CDVA of patients before and after femto-LASIK surgeries. At postoperative month 1, there was significant improvement in mean UDVA from 0.93 ± 0.23 (standard deviation) logMAR, or 20/170 (Snellen equivalent), preoperatively, to 0.40 ± 0.32, or 20/50, postoperatively (*P*=0.003). This improvement remained stable over time, with a mean UDVA of 0.44 ± 0.32, or 20/55, at postoperative month 3; 0.22 ± 0.12, or 20/33, at postoperative month 12; and 0.19 ± 0.12, or 20/31, at the last postoperative visit over 1 year. Overall, the mean UDVA at the final visit was 0.35 ± 0.29, or 20/45 (*P* < 0.0001). The preoperative UDVA ranged from 20/80 to 20/400. At final visit, 9 of 14 eyes (64.3%) had a UDVA of 20/40 or better and 12 of 14 eyes (85.7%) had a UDVA of 20/70 or better.

The results for CDVA showed no significant improvements postoperatively, but remained stable throughout the entire study period from 0.26 ± 0.19, or 20/36, preoperatively to 0.29 ± 0.26, or 20/39, at postoperative month 1 (*P*=0.47). Mean CDVA values were at 0.18 ± 0.23, or 20/30, at postoperative month 3; 0.14 ± 0.21, or 20/28, at postoperative month 12; 0.07 ± 0.09, or 20/23, at the last postoperative visit over 1 year; and 0.21 ± 0.23, or 20/32, at final visit (*P*=0.42). The preoperative CDVA ranged from 20/20 to 20/80. At final examination, 6 eyes (42.9%) improved by 1 or more Snellen lines, 5 eyes (35.7%) maintained their preoperative CDVA, and 3 eyes (21.4%) lost 1 or more Snellen lines.

### 3.3. Refractive Cylinder and Spherical Equivalent

**Figure 3:**
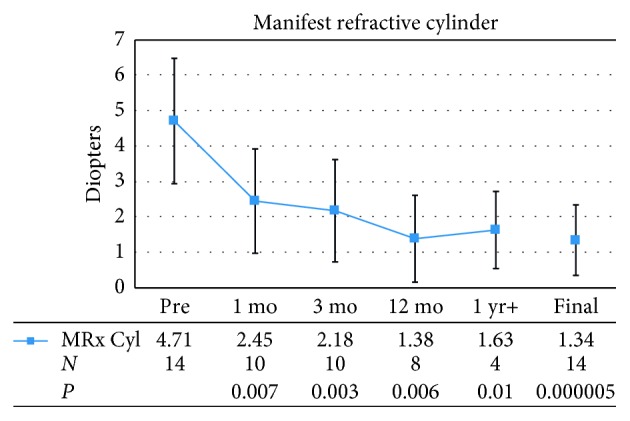
Mean preoperative (Pre) and postoperative manifest refractive astigmatism (MRx Cyl). Error bars represent one standard deviation. mo = month; yr = year; *N* = number of patients.

Figure 3 demonstrates the mean manifest refractive astigmatism of patients before and after LASIK surgeries. The preoperative refractive astigmatism ranged from 1 D to 7.25 D. At postoperative month 1, there was significant improvement in mean refractive cylinder from 4.71 ± 1.77 D preoperatively to 2.45 ± 1.47 D postoperatively (*P*=0.007). This remained stable throughout the entire study period. Mean refractive cylinder was 2.18 ± 1.45 D at postoperative month 3, 1.38 ± 1.22 D at postoperative month 12, and 1.63 ± 1.09 D at the last postoperative visit over 1 year. Taken together, the mean refractive cylinder at final visit was 1.34 ± 0.99 D (*P* < 0.0001). Complete cylindrical correction was achieved in 1 eye from a preoperative refractive astigmatism of 2.5 D. In another eye, refractive astigmatism worsened by 0.25 D, but visual acuity improved so that overall spherical equivalent was significantly improved. Cylindrical regression of 1.00 D from postoperative month 1 occurred in 1 eye, but was still an improvement at 3.25 D at final visit compared to 6.00 D preoperatively.

**Figure 4:**
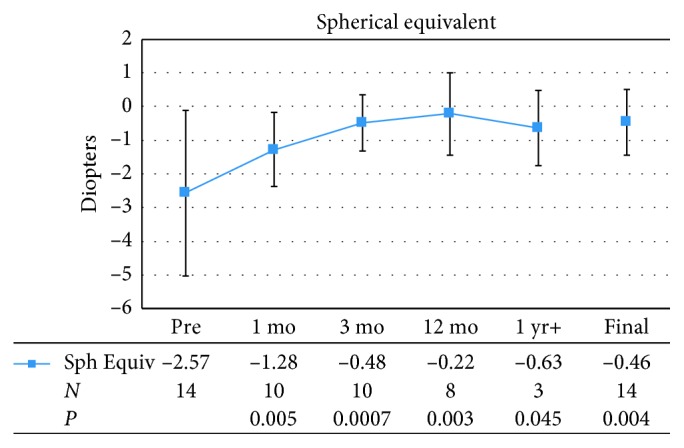
Mean preoperative (Pre) and postoperative spherical equivalent (Sph Equiv). Error bars represent one standard deviation. mo = month; yr = year; *N* = number of patients.

All fourteen eyes were myopic preoperatively, with a mean refractive spherical equivalent of −2.57 ± 2.45 D.  Figure 4 demonstrates the mean spherical equivalent of patients before and after femto-LASIK surgeries. The mean spherical equivalent improved after femto-LASIK surgery at postoperative month 1, with a mean of −1.28 ± 1.10 D (*P*=0.005). This remained stable over time with a mean of −0.48 ± 0.83 D at postoperative month 3, −0.22 ± 1.23 D at postoperative month 12, and −0.63 ± 1.11 D at the last postoperative visit over 1 year. At final visit, the mean refractive spherical equivalent was −0.46 ± 0.98 D (*P*=0.004), and 11 of 14 eyes (78.6%) were within ±1.0 D.

### 3.4. Complications

No complications arose during femto-LASIK surgery, including wound dehiscences, flap dislocations, epithelial ingrowth, flap striae, buttonholes, ectasia, or corneal edema. There were also no eyes that experienced graft infection, rejection, or failure, postoperatively. Four eyes (28.6%) underwent femto-LASIK retreatment for treatment of additional cylinder. Prior to retreatment, all four eyes had residual refractive astigmatisms of more than 1 D at postoperative month 3 of the primary femto-LASIK. After retreatment, all eyes improved favorably and without complications. Visual parameters after enhancements are included in the primary outcome results.

## 4. Discussion

In this retrospective case series, femto-LASIK has shown to be safe and effective in improving visual acuity and reducing residual astigmatism in eyes with prior FLEK. UDVA, manifest refractive astigmatism, and spherical equivalent all significantly improved by postoperative month 1 and remained stable through the final visit. While there appear to be no studies on femto-LASIK post-FLEK, there are many studies on LASIK after conventional PK with blade trephination. All of these studies have shown significant improvements in visual acuity, refractive astigmatism, and spherical equivalent, with minimal to no complications [5, 6, 18–23].

Photorefractive keratectomy was not considered in these cases due to the potential risk of haze formation, and the surgeons are not in favor of using mitomycin C on ocular surfaces and corneas that are already compromised. The use of a femtosecond laser flap allows excellent visualization of the flap and perfect centration prior to initiating the laser cut. The risk of flap dislocations and complications has been shown to be significantly reduced with femto-flaps. For these reasons, femto-LASIK is always preferred to PRK in the post-PKP patient in our practice.

The results of this unique study compare favorably with the aforementioned studies. At final examination, UDVA was 20/40 or better in 64.3% of our cases versus 29% [20], 36% [18], 54% [22], 70% [21], and 86% [19] of eyes in other studies. Statistically significant improvement in mean CDVA was not reached in this study, but overall, it remained stable from the preoperative visit to the last visit. CDVA stability is important given that femto-LASIK has been associated with loss of Snellen lines in virgin eyes [24, 25] and even seems to occur more frequently in eyes with prior PK [18–22]. In this study, 11 eyes (78.6%) either maintained or improved from the preoperative CDVA, and 21.4% of eyes lost 1 or more Snellen lines, which is similar to results reported in other studies (8% to 36%) [18–22]. The final mean astigmatism was significantly corrected from 4.71 ± 1.77 D preoperatively to 1.34 ± 0.99 D postoperatively. Mean spherical equivalent also significantly improved from −2.57 ± 2.45 D to −0.46 ± 0.98 D at final visit, and 78.6% of eyes were within ±1.0 D. This compares favorably to other studies, which have shown final spherical equivalents within ±1.0 in 50% [20], 59% [18], 73% [22], and 86% [19] of eyes.

As this was a retrospective study, follow-up data were limited given that those who were most satisfied with their improved vision often did not return as frequently as others. Furthermore, the refraction of corneal grafts can continue to change beyond 12 months; hence, longer follow-up is needed to study the long-term stability of these refractive outcomes. While this study's results suggest that femto-LASIK after FLEK is effective in improving visual acuity and reducing residual astigmatism, direct differences in surgical outcomes between femto-LASIK after FLEK and femto-LASIK after conventional PK are still unknown. However, the previous studies have shown that FLEK may have advantages postoperatively in healing, tissue alignment, and biomechanical strength compared to PKs performed with manual blade trephination [11–17]. FLEK has also been associated with less astigmatism [26] and a seven-fold increase in resistance to wound leakage [27, 28]. Therefore, it is possible that FLEK provides better graft conditions preoperatively to femto-LASIK, which may subsequently lead to improved visual outcomes and reduced complications postoperatively.

In this study, four eyes needed femto-LASIK enhancement within the first year after primary femto-LASIK. These were eyes that had preoperative cylinders high enough to require two treatments due to the limitations from laser correction. As such, these patients were told they would require at least two treatments prior to their primary LASIK operations. After retreatment, these eyes improved favorably with reduced residual refractive astigmatisms. Femto-LASIK retreatment is not unique to eyes with prior FLEK, as retreatment in post-PK corneas is known to be more common than in corneas that have not undergone any previous operations [19, 20, 23]. Enhancement rates in virgin eyes have been reported to be approximately 2–6% [24, 25, 29–32] and femto-LASIK post-PK studies have reported rates between 39% and 52.6% [19, 20, 23].

With regards to safety outcomes evaluated in this study, performing femto-LASIK surgery on eyes with prior FLEK was not associated with significantly adverse complications. Most severe complications involve damage to the transplant or to the graft-host wound interface. These adverse outcomes were not reported for any of the eyes reviewed in this study since the diameter of the femtosecond flaps was programmed to be smaller than the diameter of the graft, avoiding the graft-host junction [33, 34].

In conclusion, femto-LASIK appears to be a safe and effective surgical option to correct residual refractive error from a prior FLEK. This study is unique given the dearth of studies monitoring the performance of femto-LASIK on corneal transplants done with FS laser. The results of this study showed statistically significant improvement in UDVA, manifest refractive astigmatism, and spherical equivalent in eyes with prior FLEK. CDVA remained stable, and no surgical complications were reported. Furthermore, our results with femto-LASIK after FLEK compare favorably to results from similar studies on femto-LASIK after conventional PK. Future studies comparing direct differences between these two combinations are warranted.

## Figures and Tables

**Table 1:** Data on 14 eyes (13 patients) that underwent LASIK after FLEK.

Patient	Sex	Eye	Age at time of FLEK (years)	Primary diagnosis	Age of FLEK at time of LASIK (months)	Pre-op manifest refraction (Sph + Cyl)	Final manifest refraction (Sph + Cyl)	Need for LASIK retreatment
1	M	OS	19.2	Keratoconus	13.1	−10.25 + 6.00	−1.00 + 0.50	No
2	M	OD	55.5	Graft failure	15.2	−7.25 + 6.00	−1.50 + 1.00	No
3	M	OS	70.5	Graft failure	22.4	−7.25 + 6.00	−2.00 + 0.25	No
4	F	OS	54.7	Corneal scar	69.5	−4.75 + 3.25	−0.25 + 0.50	No
5	M	OD	42.6	Keratoconus	22.4	−6.50 + 6.00	−2.50 + 3.25	Yes
6	M	OS	62.6	Graft failure	23	−5.00 + 1.00	Plano + 1.25	No
7	F	OS	62.5	Fuchs' dystrophy	19.4	−3.00 + 6.00	−1.75 + 2.25	No
8	F	OS	71.6	Corneal scar	7.4	−6.75 + 4.25	−1.25 + 0.75	No
9	F	OS	55.8	Graft failure	48.8	−3.25 + 4.75	−2.00 + 1.00	No
10	M	OS	32.5	Graft failure	9.7	−4.75 + 3.00	−0.50 + 1.00	No
11	F	OD	77.4	Corneal scar	11.6	−1.25 + 2.50	−0.50 + 0.00	Yes
12	F	OS	17.6	Corneal scar	14.7	−1.75 + 7.25	+1.00 + 2.25	No
13	M	OD	43.8	Keratectasia	40.8	−2.50 + 4.00	−1.50 + 2.75	Yes
		OS	44.7	Keratectasia	35.5	−4.75 + 6.00	−2.00 + 2.00	Yes
Mean			50.8		25.3			

FLEK = femtosecond laser-enabled keratoplasty; LASIK = laser-assisted in situ keratomileusis; Sph = sphere; Cyl = cylinder.

## Data Availability

The data used to support the findings of this study are included within the article.
